# Supplementary material for: How does birth order influence full immunization coverage among children aged 12–23 months in India? evidence from the National Family Health Survey
Source: PLoS One. 2026 Feb 4;21(2):e0342300. doi: 10.1371/journal.pone.0342300 (PMC12871962; doi:10.1371/journal.pone.0342300)
Supplement: Attachment — Submitted filename: Plos Response.docx [file pone.0342300.s002.docx]

Dear Editor,

We are submitting our revised paper titled ***“How does Birth Order Influence Full Immunization Coverage among Children Aged 12-23 Months in India? Evidence from the National Family Health Survey”.*** We have made revisions to our paper again after paying close attention to the comments and suggestions by the editor and reviewers. We sincerely thank you for the valuable and constructive feedback that we have used to improve the quality of our manuscript.

Reviewer(s)' Comments:

Reviewer:1

This article addresses an important and policy-relevant public health issue, the influence of birth order on the full immunization coverage of children. In the context of low- and middle-income countries, such as in India where immunization coverage remains uneven and child morbidity and mortality is a persistent concern, understanding how birth order shapes immunization uptake is both timely and significant. The manuscript offers valuable insights into intra-household dynamics affecting full immunization coverage. However, there are several aspects that need further clarification to enhance the overall quality and impact of the study. Specific comments and suggestions are given below.

Abstract

In abstract include result value along with the statement such as “the likelihood of full immunization declines significantly with ….” Include OR throughout the abstract where applicable.

**Response:** *Thank you for your kind appreciation. We have edited accordingly in the revised manuscript. Please see line: 33-36.*

Introduction

Consider linking the study to relevant Sustainable Development Goals (SDGs) in introduction section to enhance the global significance of the study. The manuscript would benefit from a more robust justification of the study rationale. While the topic is relevant, the authors should better establish why this specific research is needed by engaging more thoroughly with existing literature.

**Response:** *Thank you sir/madam for your suggestions. We have revised the introduction section accordingly. Please go through lines: 67-78, 81-83.*

The stated objective of the study is “to determine how birth order influences full immunization coverage among children aged 12-23 months in India”. However, the manuscript includes spatial analysis (e.g., Moran’s I) to explore the geographic distribution or clustering of the variables, which is not reflected in the stated objectives. To maintain alignment between the study objectives and the methods employed, it is recommended that the authors revise the objective section to reflect the inclusion of spatial analysis. For instance, “Furthermore, this study also aims to explore the spatial clustering of…….”

**Response:** *We have edited accordingly in revised manuscript. Please see line: 76-78.*

Methodology

The manuscript presents maternal birth order as the main explanatory variable for full immunization coverage; however, it lacks clarity regarding independent and control variables (L: 100–102). I recommend that the authors explicitly define birth order as the key independent variable and clearly specify relevant control variables (e.g., sex of child (male and female) ……….) to account for potential confounders.

**Response:** *We have edited accordingly in the revised manuscript. Please see the line: 116-118.*

Discussion

The discussion on spatial association clusters and hotspots (L: 261–262) appears somewhat superficial. While Odisha and Chhattisgarh are identified as high-risk zones, the manuscript does not sufficiently explore the underlying causes. A more thorough integration of relevant secondary literature would enhance the depth of the explanation. While the authors provide plausible explanations for the observed association between maternal birth order and immunization coverage (L: 266-271), these explanations are not supported by relevant citations. It is therefore recommended that the authors include supportive evidence from previous national and international studies to strengthen and validate their explanations. **Response:** *Thank you, sir/madam, for your suggestions. We have revised the discussion section accordingly. Please go through lines: 297-302.*

The observed association between higher birth order and lower immunization coverage is acknowledged, yet the manuscript does not adequately discuss its policy and practical implications. I recommend that the authors briefly discuss how their findings could inform targeted policy interventions and practical strategies aimed at improving universal immunization coverage, particularly addressing the gap.

**Response:** *We have revised policy implications accordingly. Kindly go through lines: 398-404.*

In Table 1, variables such as sex of the household head, wealth index, religion, and social category are more appropriately classified as household characteristics rather than maternal characteristics. In the multilevel analysis (Table 3, Model 3), the authors should either introduce a separate model specifically for household-level characteristics or clearly integrate both maternal and household characteristics within the same model.

**Response:** *We have edited accordingly in the revised manuscript. Kindly go through revised tables 1, 2 and 3.*

Reviewer 2

The abstract effectively captures the research question and findings, but it lacks quantitative specificity. Include effect sizes or percentage differences in full immunization between birth orders to strengthen the quantitative rigor of the abstract.

**Response:** *Thank you sir/madam for your suggestion. We have edited accordingly in the revised manuscript. Kindly go through lines: 33-36.*

The rationale for exploring birth order as a determinant is sound, but the novelty could be better emphasized. Clarify how your study fills the knowledge gap, especially regarding spatial analysis of birth order and immunization in India.

**Response:** *Thank you for your kind appreciation. We have revised accordingly. Please go through line: 67-74, 76-77.*

A clearer statement of contribution will highlight the study’s uniqueness. While NFHS-5 is an appropriate and rich data source, the justification for restricting the age group to 12–23 months should be more explicit. Add a sentence justifying why this age group is ideal (e.g., global standards for “fully immunized” status).

**Response:** *Thank you sir/madam for your suggestion. We have edited accordingly in the revised manuscript. Kindly go through lines: 106-109.*

Spatial analysis using bivariate Moran’s I is appreciated but briefly described. Clarify spatial unit of analysis (districts) and provide rationale for using bivariate Moran’s I over other spatial correlation methods (e.g., GWR, Local MoIn the multilevel model section, the role of PSU (Primary Sampling Unit) as the level of clustering needs more explanation. State why PSU is the chosen cluster level and whether random intercept or random slope models were tested.

**Response:** *We have incorporated your recommendations accordingly. Please go through the statistical analysis section line: 137-145, 156-162.*

The logistic regression results are comprehensive, but effect size interpretations need refinement. When reporting odds ratios, clearly interpret them (e.g., “Children of 6th or higher birth order had 34% lower odds…”). Avoid merely reporting directionality without context.

**Response:** *We have elaborated the interpretation section. Kindly go through the lines: 245-249.*

Spatial LISA maps are referenced, but not all are described or discussed in detail. Provide more narrative interpretation of LISA outputs. What explains clusters in specific regions like Odisha or the Northeast? This would strengthen the spatial component.

**Response:** *Thank you for your valuable suggestion. We have elaborated accordingly in the revised manuscript. Kindly go through the lines: 297-302.*

The discussion includes a good synthesis of existing literature, but tends to be descriptive rather than analytical. Engage more critically with the findings. For example, why do children from the Northeast show lower coverage despite rural areas overall doing better? Discuss potential programmatic and logistical causes.

**Response:** *We have incorporated your recommendations accordingly. Please go through the discussion section line: 376-378.*

Religious and gender disparities are noted but treated cautiously. Be careful with sensitive interpretations but avoid generalisations. Back these with sociocultural or policy literature to reduce speculative tone.

**Response:** *Thank you for your suggestion.*

The limitations are mentioned but need elaboration. Clarify the potential recall bias from maternal reporting and misclassification of vaccine status. Also note the lack of longitudinal design limiting causal inference.

**Response:** *We have edited accordingly. Kindly go through line: 381-383, 384-386.*

While recommendations are included, they are somewhat generic. Offer more actionable insights—e.g., “High-parity households should be prioritized under Mission Indradhanush outreach using family-centric IEC campaigns and ASHA worker engagement.

**Response:** *Thank you for your suggestions. We have incorporated your recommendations accordingly. Please go through the conclusion section line: 398-404.*

Reference formatting is inconsistent (e.g., “[6–8]” vs. full author names in some).

**Response:** *We have revised the references as per journal guidelines.*

Tables are informative but need better captions (e.g., “Model 6 includes all covariates” could be added).

**Response:** *We have edited accordingly. Kindly go through line: 165-166.*

Consider including a robustness check (e.g., sensitivity to child sex stratification) as supplementary analysis.

**Response:** *Thank you for your insightful observation. Since our study primarily focuses upon the interplay between maternal birth order and child immunization status through the spatial lens, we have not accommodated sensitivity analysis. However, in our future study, we would definitely incorporate the same.*
