# Supplementary material for: How does birth order influence full immunization coverage among children aged 12–23 months in India? evidence from the National Family Health Survey
Source: PLoS One. 2026 Feb 4;21(2):e0342300. doi: 10.1371/journal.pone.0342300 (PMC12871962; doi:10.1371/journal.pone.0342300)
Supplement: Attachment — Submitted filename: Minor Revision Response.docx [file pone.0342300.s003.docx]

Dear Editor,

We are submitting our revised paper titled ***“How does Birth Order Influence Full Immunization Coverage among Children Aged 12-23 Months in India? Evidence from the National Family Health Survey”.*** We have made revisions to our paper again after paying close attention to the comments and suggestions by the editor and reviewers. We sincerely thank you for the valuable and constructive feedback that we have used to improve the quality of our manuscript.

Reviewer(s)' Comments:

**Reviewer:1**

I have carefully evaluated the revised version and I am pleased to note that the authors have satisfactorily addressed all the previous comments and suggestions. The manuscript is now clearly structured, the methodology is well-explained, and the results are presented coherently. The revisions have improved both the clarity and the academic quality of the paper.
I find no further issues that require modification. Therefore, I recommend the manuscript for acceptance in its current form.

**Response:** *Thank you, sir/madam, for your valuable feedback. We appreciate the recommendation for acceptance and thank the reviewer for their valuable input throughout the review process.*

**Reviewer 2**

Upon reviewing the resubmitted manuscript and the accompanying response document, it is noted that although the authors have provided written replies to each of the reviewer comments, the manuscript itself does not appear to have been revised accordingly. In several instances, the authors state that they have “addressed” the concern or “considered” the suggestion; however, the corresponding modifications are not reflected in the main text, tables, figures, or reference list. As a result, the revisions remain theoretical rather than substantive, and the manuscript in its current form does not adequately incorporate or demonstrate the improvements claimed by the authors in their response.

It is standard scholarly practice that when responding to reviewer comments, authors not only explain how each suggestion was handled but also ensure that the manuscript is visibly and systematically revised to reflect these changes. Simply providing written explanations without updating the manuscript limits the transparency of the revision process and does not allow reviewers or editors to verify whether the issues raised have been meaningfully resolved. The purpose of the revision stage is to enhance the clarity, methodological rigor, coherence, and academic contribution of the paper based on constructive peer feedback. Therefore, the absence of corresponding textual or structural changes in the manuscript is a matter of concern.

The authors are kindly advised to carefully review all comments once more and revise the manuscript in a thorough and detailed manner. Each claimed adjustment should be directly traceable in the revised text. To facilitate the review process, the authors are strongly encouraged to provide:

1. A tracked-changes version of the manuscript, clearly highlighting all modifications; and
2. A clean version of the revised manuscript.

These steps will allow the reviewers and editorial board to efficiently verify the revisions and assess the extent to which the manuscript has improved. Until the suggested changes are fully implemented in the manuscript itself, it cannot be considered satisfactorily revised.

**Response:** *We sincerely thank the reviewer for the careful reassessment of the revised manuscript and for highlighting this important concern. We have carefully re-examined all reviewer comments and have thoroughly and systematically revised the manuscript to address them in detail.*

The abstract effectively captures the research question and findings, but it lacks quantitative specificity. Include effect sizes or percentage differences in full immunization between birth orders to strengthen the quantitative rigor of the abstract.

**Response:** *Thank you, sir/madam, for your suggestion. We have edited accordingly in the revised manuscript. Kindly go through lines: 33-36.*

The rationale for exploring birth order as a determinant is sound, but the novelty could be better emphasized. Clarify how your study fills the knowledge gap, especially regarding spatial analysis of birth order and immunization in India.

**Response:** *Thank you for your kind appreciation. We have revised accordingly. Please go through line: 67-74, 76-77, 81-83.*

A clearer statement of contribution will highlight the study’s uniqueness. While NFHS-5 is an appropriate and rich data source, the justification for restricting the age group to 12–23 months should be more explicit. Add a sentence justifying why this age group is ideal (e.g., global standards for “fully immunized” status).

**Response:** *Thank you sir/madam for your suggestion. We have edited accordingly in the revised manuscript. Kindly go through lines: 106-109.*

Spatial analysis using bivariate Moran’s I is appreciated but briefly described. Clarify spatial unit of analysis (districts) and provide rationale for using bivariate Moran’s I over other spatial correlation methods (e.g., GWR, Local MoIn the multilevel model section, the role of PSU (Primary Sampling Unit) as the level of clustering needs more explanation. State why PSU is the chosen cluster level and whether random intercept or random slope models were tested.

**Response:** *We have incorporated your recommendations accordingly. Please go through the statistical analysis section line: 137-145, 156-162.*

The logistic regression results are comprehensive, but effect size interpretations need refinement. When reporting odds ratios, clearly interpret them (e.g., “Children of 6th or higher birth order had 34% lower odds…”). Avoid merely reporting directionality without context.

**Response:** *We have elaborated the interpretation section. Kindly go through the lines: 245-249.*

Spatial LISA maps are referenced, but not all are described or discussed in detail. Provide more narrative interpretation of LISA outputs. What explains clusters in specific regions like Odisha or the Northeast? This would strengthen the spatial component.

**Response:** *Thank you for your valuable suggestion. We have elaborated accordingly in the revised manuscript. Kindly go through the lines: 294-308.*

The discussion includes a good synthesis of existing literature, but tends to be descriptive rather than analytical. Engage more critically with the findings. For example, why do children from the Northeast show lower coverage despite rural areas overall doing better? Discuss potential programmatic and logistical causes.

**Response:** *We have incorporated your recommendations accordingly. Please go through the discussion section line: 380-385.*

Religious and gender disparities are noted but treated cautiously. Be careful with sensitive interpretations but avoid generalisations. Back these with sociocultural or policy literature to reduce speculative tone.

**Response:** *Thank you for your suggestion. Kindly go through line: 348-358.*

The limitations are mentioned but need elaboration. Clarify the potential recall bias from maternal reporting and misclassification of vaccine status. Also note the lack of longitudinal design limiting causal inference.

**Response:** *We have edited accordingly. Kindly go through line: 388-393.*

While recommendations are included, they are somewhat generic. Offer more actionable insights—e.g., “High-parity households should be prioritized under Mission Indradhanush outreach using family-centric IEC campaigns and ASHA worker engagement.

**Response:** *Thank you for your suggestions. We have incorporated your recommendations accordingly. Please go through the conclusion section line: 405-415.*

Reference formatting is inconsistent (e.g., “[6–8]” vs. full author names in some).

**Response:** *We have revised the references as per journal guidelines.*

Tables are informative but need better captions (e.g., “Model 6 includes all covariates” could be added).

**Response:** *We have edited accordingly. Kindly go through line: 165-166.*

Consider including a robustness check (e.g., sensitivity to child sex stratification) as supplementary analysis.

**Response:** *Thank you for your insightful observation. Since our study primarily focuses upon the interplay between maternal birth order and child immunization status through the spatial lens, we have not accommodated sensitivity analysis. However, in our future study, we would definitely incorporate the same.*
